# Hyperdynamic left ventricular ejection fraction is associated with higher mortality in COVID-19 patients

**DOI:** 10.1016/j.ahjo.2022.100134

**Published:** 2022-04-18

**Authors:** Annas Rahman, Max Ruge, Alex Hlepas, Gatha Nair, Joanne Gomez, Jeanne du Fay de Lavallaz, Setri Fugar, Nusrat Jahan, Annabelle Santos Volgman, Kim A. Williams, Anupama Rao, Karolina Marinescu, Tisha Suboc

**Affiliations:** a Department of Internal Medicine, Rush University Medical Center, Chicago, IL, United States of America; b Department of Internal Medicine, Thomas Jefferson University Hospital, Philadelphia, PA, United States of America; c Division of Cardiology, Rush University Medical Center, Chicago, IL, United States of America

**Keywords:** Left ventricular ejection fraction, Heart failure, Hyperdynamic, COVID-19, SARS-CoV-2

## Abstract

**Study objective:**

To compare the characteristics and outcomes of COVID-19 patients with a hyperdynamic LVEF (HDLVEF) to those with a normal or reduced LVEF.

**Design:**

Retrospective study.

**Setting:**

Rush University Medical Center.

**Participants:**

Of the 1682 adult patients hospitalized with COVID-19, 419 had a transthoracic echocardiogram (TTE) during admission and met study inclusion criteria.

**Interventions:**

Participants were divided into reduced (LVEF < 50%), normal (≥50% and <70%), and hyperdynamic (≥70%) LVEF groups.

**Main outcome measures:**

LVEF was assessed as a predictor of 60-day mortality. Logistic regression was used to adjust for age and BMI.

**Results:**

There was no difference in 60-day mortality between patients in the reduced LVEF and normal LVEF groups (adjusted odds ratio [aOR] 0.87, *p* = 0.68). However, patients with an HDLVEF were more likely to die by 60 days compared to patients in the normal LVEF group (aOR 2.63 [CI: 1.36–5.05]; *p* < 0.01). The HDLVEF group was also at higher risk for 60-day mortality than the reduced LVEF group (aOR 3.34 [CI: 1.39–8.42]; p < 0.01).

**Conclusion:**

The presence of hyperdynamic LVEF during a COVID-19 hospitalization was associated with an increased risk of 60-day mortality, the requirement for mechanical ventilation, vasopressors, and intensive care unit.

## Highlights

• COVID-19 patients with a hyperdynamic LVEF have poorer outcomes than patients with NLVEF or RLVEF.
• COVID-19 patients with NLVEF and RLVEF have no statistical difference in mortality or other secondary clinical outcomes.
• Hyperdynamic LVEF may serve as a useful marker of poor outcomes in patients with COVID-19.

## 1 Introduction

### 1.1 Hyperdynamic left ventricular ejection fraction

One of the most valuable measurements of cardiac function is the left ventricular ejection fraction (LVEF), as assessed by a transthoracic echocardiogram (TTE). While there has been much research about reduced LVEF, many studies investigating preserved ejection fraction usually analyze LVEF ≥ 50% as a single group. Unfortunately, this results in a limited understanding of LVEF ≥ 70%, sometimes termed hyperdynamic LVEF (HDLVEF), and any unique characteristics of this group.

Current research suggests a U-shaped survival curve when plotting the relationship between mortality and LVEF [1], [2]. One large study found that patients with an LVEF of 60–65% had the lowest mortality, and patients with higher or lower LVEF had greater mortality [1]. They also found that patients with HDLVEF had higher all-cause mortality rates regardless of a diagnosis of heart failure or inpatient/outpatient status, even after adjusting for many other confounders such as age, sex, and comorbidities [1]. Another study of intensive care unit (ICU) patients found that HDLVEF was associated with worse 28-day mortality in the critically ill than those with NLVEF [3]. The current literature suggests HDLVEF may be a pathophysiologic response to increased circulating cytokines, critical illness, and the resultant variations in physiologic parameters such as heart rate, preload, afterload, and contractility [3].

### 1.2 COVID-19 and LVEF

While coronavirus disease 2019 (COVID-19) typically targets the pulmonary system, critical cardiac manifestations have also been described. These include acute myocardial injury, arrhythmias, myocarditis, and venous thromboembolism. In heart failure, the association between COVID-19 infection and HFpEF (LVEF >50%) has been described through several pathways: inflammation, cardiac fibrosis, and direct viral infiltration, which can then unmask subclinical HFpEF or exacerbate prior history of HFpEF [4], [5], [6], [7], [8].

Prior studies have demonstrated that COVID-19 patients with decreased LVEF have worse outcomes. One small study found a higher likelihood of intubation or death in COVID-19 patients with LVEF < 55% compared to those with LVEF ≥ 55%, but this study did not differentiate between patients with a normal EF and those with a hyperdynamic EF [9]. Similarly, another study found that patients with LVEF < 40% had a significantly higher incidence of COVID-19 related hospitalization or death than LVEF ≥ 40% [10].

Although these early studies have begun to investigate the relationship between ejection fraction and various outcomes in patients with COVID-19, they usually employ a broad categorization of LVEF ≥ 50%. We believe it is worthwhile to divide that broad group, and that there is value in studying patients with HDLVEF as a separate population. Our study aims to investigate the characteristics and outcomes in COVID-19 patients with HDLVEF compared to patients with normal and reduced LVEF.

## 2 Materials and methods

### 2.1 Data and outcomes

This was a retrospective study of adult patients ≥18 years old requiring admission to the Rush University System for Health (RUSH) in Chicago, Illinois, USA, for COVID-19 infection between March and November 2020. Data such as vital signs, laboratory test values, and comorbidity history were automatically extracted from the electronic health record. Other data such as ejection fraction from TTE reports and outcomes at 30- and 60-days were manually collected from each patient chart. We followed each patient's electronic medical record for a minimum of 60 days from initial hospital admission.

After the initial data collection, only those patients who underwent a TTE during their COVID-19 admission were included. Those who met inclusion criteria were then divided into three groups: reduced LVEF (RLVEF; defined as EF < 50%), normal (NLVEF; defined as EF ≥ 50 and <70%), and hyperdynamic (HDLVEF; defined as EF ≥ 70%).

The primary outcome of this study was 60-day mortality. Secondary outcomes included: in-hospital mortality, need for intubation, vasopressors, inotropes, and admission to the intensive care unit. Additionally, the occurrence of adverse outcomes within 60 days was also assessed. These outcomes were life-threatening arrhythmias, myocardial injury (defined as cardiac troponin, cTnI, greater than the upper limit of normal), venous thromboembolism, acute heart failure exacerbation, acute kidney injury requiring renal function replacement therapy, and stroke.

### 2.2 Statistical analysis

RStudio version 1.3 (Boston, Massachusetts) was used for the statistical analysis of this study. To generate a Kaplan-Meier plot and survival estimates, the survival and survminer packages were used, respectively.

If normally distributed, continuous variables were summarized with mean and standard deviation. If not normally distributed, then median and interquartile ranges were reported. Finally, categorical variables were described with counts and percentages. *t*-Tests were used to compare continuous variables, and Pearson chi-square tests were performed for categorical variables.

Multivariable logistic regression was performed using the ejection fraction group as a predictor for primary and secondary variables. All logistic regression models were adjusted for age and body mass index. These are reported with odds ratios (OR) and 95% confidence intervals (CI). For all statistical tests, the threshold for significance was set to a *p*-value < 0.05.

## 3 Results

**Fig. 1:**
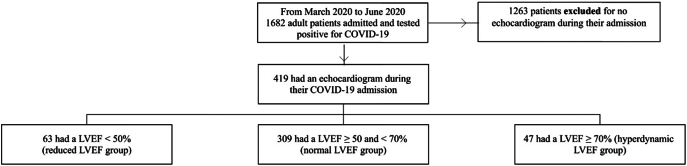
Study cohort selection diagram.

Of the initial 1682 hospitalized COVID-19 patients, 419 (24.9%) met the inclusion criteria by having a TTE during their admission (Fig. 1). 1263 patients were excluded for not having a TTE. Of the 419 patients in our cohort, the median LVEF was 57.5% with an interquartile range of 12.3%, and minimum and maximum values of 12.5% and 78.0% (Supplemental Fig. 1). 63 (15.0%) patients were in the RLVEF group, 309 (73.4%) in the NLVEF, and 47 (11.2%) in the HDLVEF group with median ejection fractions of 37.5%, 57.5%, and 72.5%, respectively.

The median age of our cohort was 63 years old with an interquartile range of 22; no significant age differences were present between the groups. Those in the HDLVEF and RLVEF groups were more likely to be African American (Table 1).

**Table 1:** Baseline characteristics of each ejection fraction group.

	Reduced LVEF	Normal LVEF	Hyperdynamic LVEF	p-Value
n	63	309	47	
Age (median, IQR)	63.00 [53.00, 75.00]	63.00 [51.00, 73.00]	63.00 [48.00, 69.50]	0.616
Male (%)	33 (52.4)	172 (55.7)	29 (61.7)	0.617
BMI (median, IQR)	29.20 [24.95, 36.00]	30.40 [27.02, 36.70]	31.60 [27.60, 35.95]	0.287
Race (%)				0.002
White	18 (30.0)	92 (31.2)	7 (14.9)	
Other	13 (21.7)	120 (40.7)	19 (40.4)	
African American	29 (48.3)	83 (28.1)	21 (44.7)	
Comorbidities				
Current smoker (%)	2 (3.6)	15 (5.5)	2 (5.4)	0.846
Atrial fibrillation (%)	28 (44.4)	96 (31.1)	7 (14.9)	0.004
Coronary artery disease (%)	34 (54.0)	129 (41.7)	16 (34.0)	0.090
Hypertension (%)	50 (79.4)	231 (74.8)	35 (74.5)	0.732
Chronic kidney disease (%)	29 (46.0)	110 (35.6)	15 (31.9)	0.225
COPD (%)	9 (14.3)	31 (10.0)	6 (12.8)	0.565
Diabetes mellitus (%)	31 (49.2)	178 (57.6)	28 (59.6)	0.428
Asthma (%)	10 (15.9)	40 (12.9)	4 (8.5)	0.521
Cancer (%)	12 (19.0)	41 (13.3)	4 (8.5)	0.265
Ventricular arrhythmia (%)	13 (20.6)	27 (8.7)	5 (10.6)	0.021
Stroke (%)	16 (25.4)	70 (22.7)	7 (14.9)	0.394
Acute myocardial infarction (%)	25 (39.7)	92 (29.8)	14 (29.8)	0.295
DVT or pulmonary embolism (%)	18 (28.6)	84 (27.2)	19 (40.4)	0.175
Initial labs (median, IQR)				
Troponin (cTnI)	0.12 [0.02, 0.44]	0.03 [0.01, 0.10]	0.03 [0.01, 0.08]	<0.001
White blood cell count	7.65 [5.50, 11.14]	7.64 [5.80, 10.87]	8.61 [6.21, 13.99]	0.204
Lymphocyte number	1.11 [0.62, 1.65]	0.96 [0.70, 1.36]	0.95 [0.64, 1.27]	0.342
Hemoglobin	13.30 [11.45, 14.30]	12.80 [11.10, 14.40]	12.50 [11.00, 14.20]	0.671
Platelet count	210.00 [165.00, 274.50]	210.00 [161.00, 288.00]	219.00 [193.00, 257.50]	0.402
Creatinine	1.52 [1.04, 2.25]	1.09 [0.86, 1.85]	1.25 [0.85, 1.83]	0.029
CRP	111.00 [33.50, 198.90]	119.75 [45.80, 199.25]	202.20 [102.75, 283.75]	0.003
Ferritin	613.70 [295.00, 1564.00]	818.32 [356.88, 1616.00]	1211.00 [293.00, 1991.00]	0.520
LDH	466.00 [333.00, 780.00]	435.00 [297.75, 570.25]	569.50 [400.25, 720.50]	0.004
ESR	37.00 [15.00, 95.00]	60.00 [35.00, 80.00]	55.00 [45.75, 82.50]	0.327
Vital signs (median, IQR)				
Systolic BP	130.00 [115.00, 155.00]	129.00 [114.00, 147.00]	140.00 [125.00, 148.50]	0.147
Diastolic BP	76.00 [66.00, 91.50]	74.00 [65.00, 85.00]	78.00 [65.00, 90.50]	0.311
Heart rate	104.00 [85.50, 118.00]	98.00 [85.00, 112.00]	99.00 [88.50, 112.50]	0.578
Respiratory rate	20.00 [18.00, 24.00]	21.00 [18.00, 26.00]	22.00 [20.00, 30.50]	0.024
Pulse oximetry	96.00 [91.50, 98.00]	94.00 [87.00, 97.00]	90.00 [82.00, 96.00]	0.006
Temperature	98.40 [97.60, 99.45]	99.00 [98.00, 100.10]	98.70 [97.95, 100.10]	0.065

IQR = interquartile range; BMI = body mass index; CKD = chronic kidney disease; COPD = chronic obstructive pulmonary disorder; DVT = deep venous thrombosis; CRP = c-reactive protein; LDH = lactate dehydrogenase; BP = blood pressure.

The RLVEF group had higher pre-existing diagnoses of atrial fibrillation and ventricular arrhythmias when comparing comorbidities. Otherwise, there were no differences between the groups in the proportion of other medical conditions present before hospital admission, including a history of CAD, HTN, CKD, diabetes mellitus, asthma, or cancer.

When comparing initial lab values, the RLVEF group had higher initial cardiac troponin I and creatinine levels than the other groups, while the HDLVEF group had significantly higher initial CRP and LDH values. There was no difference between the three groups in WBC count, lymphocyte number, hemoglobin, platelet count, ESR, or ferritin.

In comparing initial vital signs, the HDLVEF cohort had a higher respiratory rate and a lower oxygen saturation than the RLVEF and NLVEF groups. There was no difference in systolic or diastolic blood pressures, heart rate, or temperature.

A separate comparison, not part of the main analysis, was done to compare the patients who met the study inclusion criteria with the patients that were excluded – patients admitted with COVID-19 infection but did not have an echocardiogram during admission. The echocardiogram group had a significantly increased incidence of most comorbidities, including atrial fibrillation, coronary artery disease, hypertension, and chronic kidney disease (Supplemental Table 1). The incidence of both 60-day mortality (25.1% vs 7.7%, *p* < 0.001) and severe infection (72.3% vs 27.9%, p < 0.001) was also higher in those who had an echocardiogram versus those who did not.

**Fig. 2:**
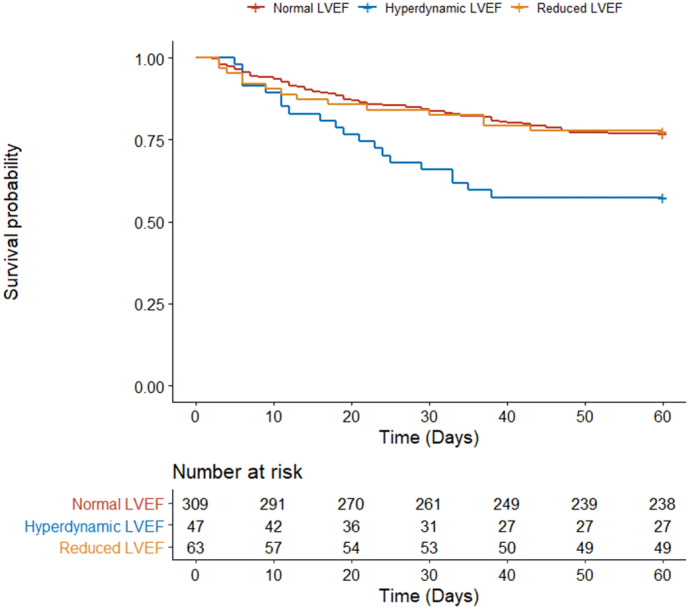
Kaplan-Meier survival estimates comparing mortality in COVID-19 patients with normal vs. hyperdynamic left ventricular ejection fractions.

**Fig. 3:**
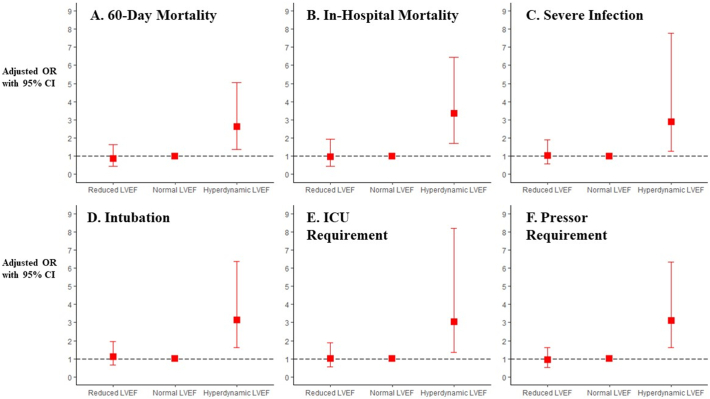
Multivariable odds ratios, adjusted for age and body mass index, with 95% confidence intervals of primary (A) and secondary (B, C, D, E, and F) outcomes. OR = odds ratio; CI = confidence interval; ICU = intensive care unit.

The 60-day mortality rate in the RLVEF, NLVEF, and HDLEF groups was 22.2%, 23.0%, and 42.6%, respectively (Fig. 2). Those in the HDLVEF group were significantly more likely to suffer 60-day mortality than those in the RLVEF (adjusted OR [aOR] 3.34 [CI: 1.39–8.42]; *p* < 0.01) or the NLVEF groups (aOR 2.63 [CI: 1.36–5.05]; p < 0.01; Fig. 3A, Table 2). The RLVEF group was no more likely to suffer 60-day mortality than the NLVEF group (aOR 0.87 [CI: 0.44–1.65]; *p* = 0.68).

**Table 2:** Primary and secondary outcomes of each ejection fraction group.

	Reduced LVEF			Normal LVEF			Hyperdynamic LVEF		
	Incidence	aOR (95% CI)	p-Value	Incidence	aOR (95% CI)	p-Value	Incidence	aOR (95% CI)	p-Value
Primary outcome									
60-Day mortality	22.2%	0.87 (0.44–1.65)	0.68	23.0%	Ref	–	42.6%	2.63 (1.36–5.05)	<0.01
Secondary outcomes									
Severe infection	71.4%	1.02 (0.57–1.90)	0.94	70.2%	Ref	–	87.2%	2.89 (1.27–7.79)	<0.05
Intubation	47.6%	1.12 (0.65–1.94)	0.68	45.0%	Ref	–	72.3%	3.13 (1.62–6.37)	<0.01
ICU requirement	69.8%	1.02 (0.57–1.88)	0.95	68.9%	Ref	–	87.2%	3.04 (1.34–8.20)	<0.05
Pressor requirement	42.9%	0.93 (0.53–1.61)	0.79	45.0%	Ref	–	72.3%	3.11 (1.61–6.34)	<0.01

LVEF = left ventricular ejection fraction; aOR = adjusted odds ratio; CI = confidence interval.

For secondary outcomes, the HDLVEF was at greater risk for in-hospital mortality than the NLVEF group (aOR 3.35 [CI: 1.72–6.46]; *p* < 0.001), while those with RVLEF were at no higher risk (aOR 0.97 [CI: 0.45–1.93]; p – 0.93; Fig. 3B). The HDLVEF was also at greater risk of in-hospital mortality than the RLVEF group (aOR 3.70 [CI: 1.52–9.58]; *p* < 0.01).

When compared to the NLVEF group, HDLVEF patients were also more likely to suffer severe infection (aOR 2.89 [CI: 1.27–7.79]; *p* < 0.05) or require intubation (aOR 3.13 [CI: 1.62–6.37]; p < 0.01), the intensive care unit (aOR 3.04 [CI: 1.34–8.20]; p < 0.05), or vasopressors (aOR 3.11 [CI: 1.61–6.34]; p < 0.01) (Fig. 3, Table 2). No difference was present when comparing the incidence of these secondary outcomes between the RLVEF group and the NLVEF group.

When comparing the incidence of 60-day major adverse cardiovascular events (MACE), the RLVEF was most likely to suffer at least one event (66.7%), followed by the HDVLEF group (51.1%) and then the NLVEF group (38.5%) (Table 3). The RLVEF was more likely to have a myocardial injury (*p* < 0.001) and the signs and symptoms of a heart failure exacerbation (p < 0.001) than the NLVEF and HDLVEF groups. The NLVEF group was significantly less likely to have a deep venous thrombosis (*p* < 0.05), while the HDLVEF group was most likely to have an acute kidney injury requiring renal replacement therapy (*p* < 0.01).

**Table 3:** Major adverse cardiovascular events by left ventricular ejection fraction group.

	NLVEF	HDLVEF	RLVEF	*p*-Value
n	309	47	63	
Total MACE (%)	119 (38.5)	24 (51.1)	42 (66.7)	<0.001
Myocardial injury (%)	18 (5.8)	3 (6.4)	13 (20.6)	<0.001
Stroke (%)	2 (0.6)	0 (0.0)	0 (0.0)	0.699
Life-threatening arrhythmia (%)	38 (12.3)	10 (21.3)	10 (15.9)	0.222
DVT (%)	17 (5.5)	7 (14.9)	7 (11.1)	0.034
HF exacerbation (%)	10 (3.2)	1 (2.1)	21 (33.3)	<0.001
Requiring RRT (%)	41 (13.3)	15 (31.9)	14 (22.2)	0.003
PE (%)	26 (8.4)	6 (12.8)	2 (3.2)	0.177

NLVEF = normal left ventricular ejection fraction; HDLVEF = hyperdynamic left ventricular ejection fraction; RLVEF = reduced left ventricular ejection fraction; ICU = intensive care unit; MACE = major adverse cardiac events; DVT = deep vein thrombosis; HF = heart failure; RRT = renal replacement therapy; PE = pulmonary embolism.

## 4 Discussion

### 4.1 COVID-19 infection and LVEF

In this retrospective cohort study of 419 patients who underwent a TTE during their COVID-19 admission, patients with a HDLVEF, but not those with a RLVEF, were at an increased risk for both in-hospital and 60-day mortality. Additionally, only the HDLVEF group was at increased risk for other markers of severe COVID-19 infection, including mechanical ventilation, vasopressor requirement, and need for ICU. This finding supports and extends prior data showing increased mortality with HDLVEF [3].

Differences in pathophysiology might explain the difference in mortality between the HDLVEF and RLVEF groups demonstrated in this study. RLVEF is caused by decreased myocardial contractility, which is often ischemic in etiology and can lead to maladaptive remodeling when chronic. In contrast, the pathophysiology of HDLVEF is not entirely understood. In severe illness, the cardiac systolic function is variable and is related to multiple parameters, including heart rate, preload, afterload, and contractility [3], [11]. HDLVEF can be seen in septic states where low systemic vascular resistance and increased circulating catecholamines lead to increased contractility [12]. Mismatch of myocardial contractility and arterial compliance, ventriculoatrial decoupling in critical illness, and diastolic dysfunction have also been theorized to play a role [13], [14]. Thus, HDLVEF may represent a physiologic inflammatory response in COVID-19. In this study, the HDLVEF group had higher initial CRP and LDH levels (Table 1), while RLVEF resulted from a chronic process already present at the time of infection and was less affected. This relationship is consistent with what other researchers have hypothesized, that COVID-19 may cause new HFpEF, unmask subclinical HFpEF, or exacerbate existing HFpEF [5]. Although these determinations are best left for future research since our study was not able to compare echocardiograms prior to admission for COVID-19 infection.

Overall, the hyperdynamic LVEF is more likely to be a response to inflammation or infection rather than pre-existing cardiac dysfunction. This was supported by higher baseline CRP, LDH, respiratory rate, and lower oxygen saturation in the HDLVEF group compared to the RLVEF and NLVEF groups (Table 1). And while the RLVEF group did have higher cardiac troponin and creatinine, the overall analysis showed that hyperdynamic left ventricular function was associated with the primary and secondary outcomes (60-day mortality, severe infection, intubation, ICU requirement, pressor requirement) (Table 2).

In prior studies of patients without COVID-19, both RLVEF and HDLVEF have been associated with higher mortality. In a study of ICU non-COVID-19 patients with HDLVEF, Paonessa et al. found that HDLVEF was associated with increased 28-day mortality compared to patients with a NLVEF [3]. That study also found that HDLVEF was associated with female sex, increased age, and history of hypertension or cancer, which we did not find in our study cohort. Male sex has been associated with a more severe course in COVID-19 and may be a factor in the COVID-19 population [15], which we found in our larger study cohort and previously reported [16]. In heart failure with reduced ejection fraction, mortality increases linearly as LVEF decreases [2], [17], [18]. The higher mortality of RLVEF and HDLVEF results in a U-shaped curve that diverges from normal LVEF values [1], [2], [19]. Our study findings differ because outcomes in the RLVEF cohort did not significantly differ from those in the NLVEF cohort. (Fig. 3).

Although our study cohort showed a high 60-day mortality rate of 25.1%, this is within previously reported mortality rates of 4–28% for COVID-19 patients [20]. The mortality rate of our population was towards the upper end of this range, likely because our study included only patients with TTEs done during admission, which was an inherently sicker population with higher rates of most comorbidities, severe illness, and mortality (Supplemental Table 1).

### 4.2 Limitations

Our study is limited by its retrospective nature and the extraction of data from electronic medical records. Variables not captured in the database may be confounding variables, and causation cannot be inferred between predictors and outcomes.

Furthermore, our study cohort of patients who had TTEs during their COVID-19 hospitalization represents an overall sicker group than the general population. In our separate analysis comparing the patients included in the study with those excluded (i.e., those who had a TTE during their admission and those who did not), we found that our included patient population had significantly more comorbidities, was older, had higher 60-day mortality, and more severe infection than the excluded group (Supplemental Table 1). Most patients in our initial population were excluded due to a lack of TTE. Although the included population was sicker as a whole, by dividing that group into three discrete LVEF groups, we find that the HDLVEF cohort had the worst outcomes – a valuable finding since many studies do not pay particular attention to hyperdynamic LVEF.

Comorbidities are mainly based on ICD-10 codes, which could be a source of inconsistent reporting, but this may be mitigated by including only a single health care provider system. Furthermore, discharged patients were presumed to have favorable outcomes, and readmission rates were only captured if the patient returned to our hospital system or a Chicago-area hospital that used our same electronic medical record.

Finally, one of the limitations may be in the definition of HDLVEF itself since not all studies use the same standard. Many researchers study LVEF using various 5–10% intervals or LVEF ≥ 70% to describe hyperdynamic ejection fractions. And while the European Society of Cardiology does not have a formal definition of HDLVEF, the American College of Cardiology defines it as >70% [21]. LVEF measurement can be subjective, and studies like ours show that LVEF ≥ 70% is associated with different outcomes than patients with LVEF < 70%.

## 5 Conclusions

This study analyzed the characteristics and outcomes of COVID-19 patients by comparison of LVEF. We were specifically interested in understanding patients with a hyperdynamic ejection fraction. Compared to NLVEF or RLVEF, patients with HDLVEF admitted with COVID-19 were more likely to have increased 60-day mortality and severe infection.

## Funding

The authors received no financial support for the research, authorship, or publication of this manuscript.

## Declaration of competing interest

The authors declare that they have no known competing financial interests or personal relationships that could have appeared to influence the work reported in this paper.

## Appendix A Supplementary data

Supplementary material Image 1
