# Supplementary material for: Hyperdynamic left ventricular ejection fraction is associated with higher mortality in COVID-19 patients
Source: Am Heart J Plus. 2022 Apr 18;14:100134. doi: 10.1016/j.ahjo.2022.100134 (PMC9013697; doi:10.1016/j.ahjo.2022.100134)
Supplement: Supplementary file 1 — Supplementary material [file mmc1.docx]

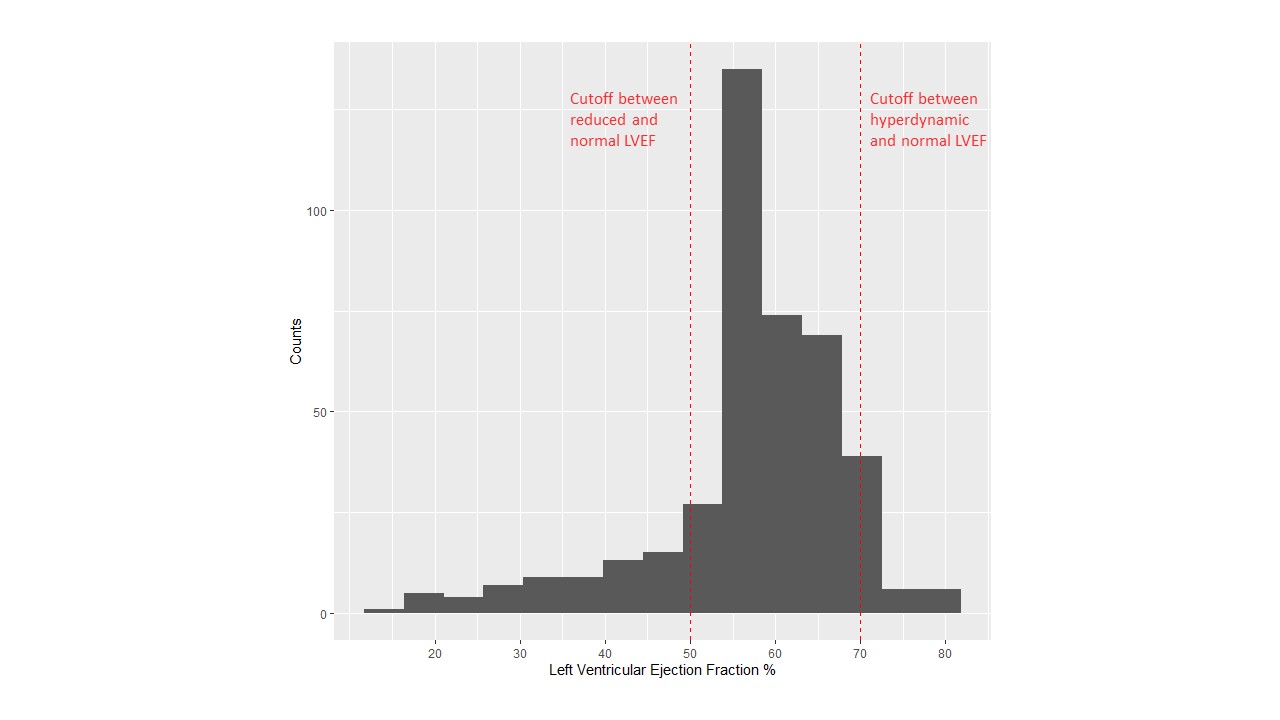


**Supplemental Figure 1.** Histogram plot of the frequency of left ventricular ejection fractions

LVEF = left ventricular ejection fraction

**Supplemental Table 1.** Comparison of baseline characteristics of those who had an echocardiogram versus those who did not

|  | Echocardiogram | No Echocardiogram | p-value |
| --- | --- | --- | --- |
|  |  |  |  |
| n | 419 | 1263 |  |
| Age (median, IQR) | 63.00 [51.00, 73.00] | 57.00 [45.00, 69.50] | <0.001 |
| Male (%) | 234 (55.8) | 682 (54.0) | 0.547 |
| BMI (median, IQR) | 30.40 [26.60, 36.70] | 31.00 [26.30, 37.10] | 0.751 |
| Race (%) |  |  | 0.242 |
| White | 117 (29.1) | 333 (28.1) |  |
| Other | 152 (37.8) | 407 (34.3) |  |
| African American | 133 (33.1) | 446 (37.6) |  |
| Comorbidities |  |  |  |
| Current Smoker (%) | 19 ( 5.2) | 58 ( 5.0) | 0.952 |
| Atrial Fibrillation (%) | 131 (31.3) | 123 ( 9.7) | <0.001 |
| Coronary Artery Disease (%) | 179 (42.7) | 267 (21.1) | <0.001 |
| Hypertension (%) | 316 (75.4) | 758 (60.0) | <0.001 |
| Chronic Kidney Disease (%) | 154 (36.8) | 269 (21.3) | <0.001 |
| COPD (%) | 46 (11.0) | 90 ( 7.1) | 0.016 |
| Diabetes Mellitus (%) | 237 (56.6) | 537 (42.5) | <0.001 |
| Asthma (%) | 54 (12.9) | 181 (14.3) | 0.511 |
| Cancer (%) | 57 (13.6) | 155 (12.3) | 0.531 |
| Ventricular Arrhythmia (%) | 45 (10.7) | 34 ( 2.7) | <0.001 |
| Stroke (%) | 93 (22.2) | 154 (12.2) | <0.001 |
| Acute Myocardial Infarction (%) | 131 (31.3) | 128 (10.1) | <0.001 |
| DVT or Pulmonary Embolism (%) | 121 (28.9) | 124 ( 9.8) | <0.001 |
| Outcomes |  |  |  |
| 60-Day Mortality (%) | 105 (25.1) | 97 ( 7.7) | <0.001 |
| Severe Infection (%) | 303 (72.3) | 353 (27.9) | <0.001 |

IQR = interquartile range; BMI = body mass index; CKD = chronic kidney disease; COPD = chronic obstructive pulmonary disorder; DVT = deep venous thrombosis
